# An Interesting Case

**Published:** 1892-06

**Authors:** J. Emmett Blackshear


### AN INTERESTING CASE.

Mr. J. V., a young man twenty-one years of age, was first seen July 5, 1891. Had been for eight months under treatment of syphilis, and all the while growing worse. Was very much prostrated, and was suffering with an immense abscess on the outer portion of the upper third of the left thigh; also one on the left side of the neck. Had, moreover, a large tumor on the right side of the neck and a still larger one on the back, both tending, of course, to suppuration. Prescribed verrhus clemiana, two teaspoonfuls four times a day, to be increased in two weeks to four teaspoonfuls.

At the expiration of two weeks, the abscess on thigh was discharging profusely, general appearance of patient improved, and he reported himself as feeling much better. Ordered the dose increased gradually to six teaspoonfuls, four times daily.

At the expiration of six weeks the abscess on thigh was still discharging freely; the one on neck scarcely at all. Ordered the suspension of the medicine for a week, during which time the discharge from abscess on thigh diminished perceptibly; the one on the neck ceased entirely. On resuming the medicine, the discharges from both abscesses became again profuse, and so continued for about two weeks longer, when it began to subside; the tumors began to diminish in size, and in four months from the time the treatment was commenced, all symptoms of the disease had disappeared. Advised him however, to continue the medicine a few months longer.

J. EMMETT BLACKSHEAR, M. D.

